# Effects of Astaxanthin as a Feed Additive on Growth Performance, Intestinal Microbiota and Clinical Parameters in Preweaning Female Holstein Calves: A Preliminary Study

**DOI:** 10.3390/ani16081173

**Published:** 2026-04-11

**Authors:** Elena Scaglia, Valeria Sergi, Laura Giagnoni, Livio Galosi, Anna Simonetto, Giulia Ferronato, Gianni Gilioli, Valentina Caprarulo

**Affiliations:** 1 Department of Civil, Environmental, Architectural Engineering and Mathematics, Università di Brescia, 25121 Brescia, Italy; valeria.sergi@unibs.it (V.S.); laura.giagnoni@unibs.it (L.G.); anna.simonetto@unibs.it (A.S.); giulia.ferronato@unibs.it (G.F.); gianni.gilioli@unibs.it (G.G.); valentina.caprarulo@izsler.it (V.C.); 2 School of Biosciences and Veterinary Medicine, University of Camerino, 62024 Matelica, Italy; livio.galosi@unicam.it; 3 Istituto Zooprofilattico Sperimentale della Lombardia e dell’Emilia Romagna “Bruno Ubertini”, 25124 Brescia, Italy

**Keywords:** dairy calves, calves’ diarrhea, dairy farm, gut health, preweaning phase, antioxidants, feed additives

## Abstract

The preweaning period is a critical phase for dairy calves, during which gastrointestinal disorders, particularly diarrhea, remain a major cause of morbidity and antimicrobial use. Astaxanthin, a xanthophyll carotenoid with antioxidant and anti-inflammatory properties, has shown potential to support intestinal health in several animal species, but information in preweaning dairy calves is limited. The objective of this study was to evaluate the effects of astaxanthin supplementation administered through milk replacer on growth performance, clinical health, metabolic profile, and fecal microbiota in preweaning Holstein calves. Twenty-four female Holstein calves (body weight, mean ± SD: 49.51 ± 12.14 kg) were randomly assigned to a control group (CTR; *n* = 12) or an astaxanthin-supplemented group (TRT; *n* = 12). Treated calves received 40 mg/d of astaxanthin from week 0 to 4 and 80 mg/d from week 4 to 8. Body weight, feed intake, rectal temperature, and fecal consistency score, fecal color score and clinical scores were recorded throughout the trial. Blood samples were collected in weeks 0 and 8 for metabolic profiling, and fecal samples were collected in weeks 0, 4, and 8 to assess fecal consistency and bacterial populations. Astaxanthin supplementation did not affect body weight, average daily gain, or overall feed intake. However, treated calves exhibited a lower frequency of abnormal fecal consistency scores, indicating reduced diarrhea incidence compared with control calves. Fecal microbiological analysis revealed lower coliform counts in astaxanthin-supplemented calves in weeks 4 and 8, whereas total bacterial counts were greater in week 8. Most blood metabolites were primarily influenced by age-related physiological changes; however, circulating calcium concentrations were greater in treated calves. These results suggest that astaxanthin may represent a promising nutritional strategy to support gastrointestinal health during early life, although larger studies are needed to confirm these findings.

## Simple Summary

The neonatal period is a critical phase for dairy calves, as they are particularly susceptible to health problems and environmental stress. For this reason, nutritional strategies that support calf health are of increasing interest. Astaxanthin is a natural compound known for its antioxidant properties and its potential role in supporting physiological functions. This study evaluated the effects of adding astaxanthin to milk replacer in pre-weaned dairy calves. Growth performance, feed intake, health indicators, and selected blood parameters were monitored during the experimental period. The results showed that astaxanthin supplementation did not negatively affect calf performance and maintained normal physiological conditions, while reducing the occurrence of diarrhea and modulating the fecal microbiota. These findings suggest that astaxanthin may represent a promising nutritional strategy to support calf health during early life in dairy production systems.

## 1. Introduction

The preweaning period is a critical phase in the life of dairy calves, characterized by rapid growth, maturation of the immune system, and the establishment of physiological and microbial homeostasis [1]. During this stage, calves are particularly susceptible to enteric and respiratory diseases, which remain the leading causes of morbidity and mortality in early life. Health disorders occurring in this period have been consistently associated with impaired growth performance, reduced feed efficiency, alterations in mammary gland development, and decreased lifetime milk yield [2]. Beyond their long-term productive consequences, poor calf health negatively affects the environmental sustainability of dairy systems by increasing resource use, medical inputs, and waste outputs per unit of milk produced. Collectively, these challenges underscore the need for targeted nutritional strategies aimed at enhancing calf health and resilience while reducing the reliance on antibiotic treatments [3,4].

In recent years, nutritional strategies aimed at supporting gastrointestinal health and reducing reliance on antibiotics have gained increasing attention. Functional feed additives, including antioxidants, probiotics, and plant- or algae-derived bioactive compounds, have been proposed as promising tools to enhance intestinal resilience, modulate immune responses, and improve health outcomes in young ruminants [3,4,5]. Among these compounds, astaxanthin, a xanthophyll carotenoid naturally synthesized by microalgae such as *Haematococcus pluvialis*, has emerged as a potent antioxidant with documented anti-inflammatory and immunomodulatory properties [6,7,8].

Astaxanthin exhibits a unique molecular structure that enables efficient scavenging of reactive oxygen species and stabilization of cellular membranes, conferring greater antioxidant capacity than several conventional antioxidants, including vitamins E and C [8]. Beyond its antioxidant activity, astaxanthin has been shown to modulate key inflammatory pathways, such as NF-κB and MAPK signaling, resulting in reduced expression of pro-inflammatory cytokines and enhanced immune function [6,9]. Moreover, astaxanthin enhances both innate and adaptive immune responses by promoting macrophage activity, antibody production, and natural killer cell function [6,10]. Growing evidence suggests also that astaxanthin can influence intestinal barrier integrity and gut microbial ecology, either directly through antibacterial activity or indirectly by reducing oxidative stress and inflammation within the intestinal mucosa [7,10,11,12].

Consistent with these mechanisms, dietary astaxanthin supplementation has produced favorable effects on health, antioxidant status and intestinal function in several monogastric species, including poultry, swine, and aquaculture models. Studies in poultry [7], swine [8], and aquaculture models [11] report improved growth performance and feed efficiency. In addition, these studies report increased activity of endogenous antioxidant enzymes such as superoxide dismutase, catalase, and glutathione peroxidase [10,13]. In adult ruminants, preliminary studies suggest potential benefits of astaxanthin on reproductive performance, stress resilience, and inflammatory responses, although its bioavailability may be limited by ruminal degradation [8,14]. In contrast, preweaning calves represent a unique physiological model in which ruminal function is not yet fully developed, potentially allowing dietary carotenoids to escape extensive microbial degradation and be absorbed more efficiently, similarly to monogastric species. Despite the growing interest in astaxanthin as a functional feed additive, information regarding its effects in preweaning dairy calves remains limited, particularly with respect to gastrointestinal health and fecal microbiota. Given the central role of intestinal dysbiosis in the pathogenesis of neonatal calf diarrhea and the increasing need for non-antibiotic nutritional interventions, evaluating the potential of astaxanthin to support gut health during early life is of relevance.

Therefore, the objective of this study was to evaluate the effects of astaxanthin supplementation administered through milk replacer on growth performance, clinical health, metabolic profile, and fecal microbiota in preweaning Holstein calves. We hypothesized that astaxanthin supplementation would reduce diarrhea incidence and modulate fecal bacterial populations without negatively affecting growth performance during the preweaning period.

## 2. Materials and Methods

The trial was performed from June to October 2024 (T° min 10.8–18.5 °C, max 18.1–28.6 °C) at a commercial dairy farm located in the province of Brescia (Lombardy region, Italy). The experimental protocol was approved by Animal Welfare Body of the University of Camerino (approval number 2/2025).

### 2.1. Animal Management and Experimental Design

**Figure 1:**
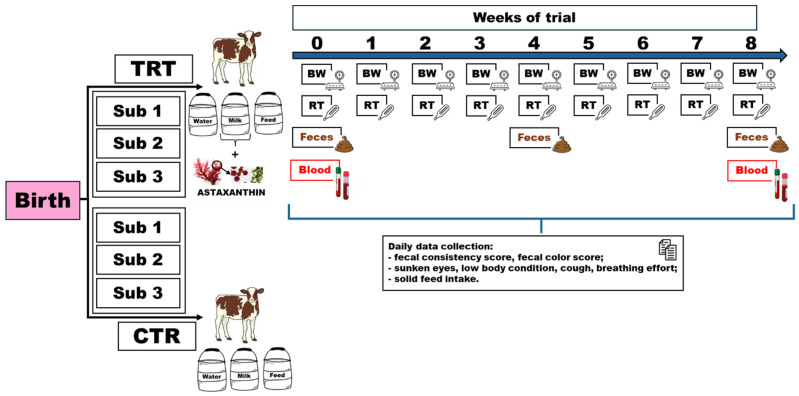
Experimental design and timeline of the trial illustrating calf groups, subgroups, diet, daily and weekly data collection, feces and blood collection. Calf groups: treatment group (TRT) and control group (CTR). Sub indicates the subgroup, which is the same for each group. Sub 1: three calves per treatment with 1–2 days of life. Sub 2: three calves per treatment with 3–4 days of life. Sub 3: six calves per treatment with 5–7 days of life. Weekly evaluations: body weight (BW) and rectal temperature (RT). Feces were collected in week 0, 4, 8 and blood samples in week 0 and 8.

A total of 24 Holstein female calves were randomly assigned to one of two treatment groups: TRT (treatment group, 12 calves; astaxanthin supplementation) or CTR (control group, 12 calves, no supplementation). Within each treatment group, calves were further stratified by age at supplementation initiation into three subgroups categories (Sub): 1–2, 3–4, and 5–7 days of age, with 4 calves per treatment × age combination. This 2 × 3 factorial design allowed assessment of the effects of treatment, age at supplementation initiation, and their interaction on calf responses. Random allocation within age strata minimized potential confounding effects (Figure 1).

Immediately after birth animals were housed in individual straw-bedded boxes. The calves were provided with 4 L of high-quality colostrum (>25° Brix) via an esophageal feeder within six hours of birth. The colostrum was obtained from the colostrum bank. Thereafter, the animals were fed 4 L of milk replacer (MR) twice daily (UVL srl, Roè Volciano, Brescia, Italy; Table 1), with the volume increased by 1 L per week until a maximum intake of 8 L per day was reached. The MR powder (140 g/L) was reconstituted in water at 39 °C and provided to the animals in buckets. The treatment group (TRT) received 40 mg/day of astaxanthin (UVL srl, Roè Volciano, Brescia, Italy) from week 0 to 4 and 80 mg/day from week 5 to 8 of the trial. The inclusion level of the additive was determined by the manufacturer. The supplement was administered via the morning milk replacer (MR), which was homogenized using a whisk prior to feeding. No milk refusals were observed throughout the experimental period. Throughout the entire trial, all animals had ad libitum access to water and calf starter (SF, UVL srl, Roè Volciano, Brescia, Italy; Table 1). The refusal of calf feed on a daily basis was recorded for the purpose of evaluating average daily feed intake (ADFI).

### 2.2. Measurements, Sampling and Analysis

Body weight (BW) and rectal temperature (RT) were individually and weekly monitored on weeks 0, 1, 2, 3, 4, 5, 6, 7, and 8 of trial before the MR meal distribution.

The clinical health of the subjects was evaluated using a standardized scoring system encompassing several parameters: sunken eyes (0–4), body condition (0–5), cough (0–2), and breathing effort (0–1) [15]. Sunken eyes were subjected to a visual assessment, and confirmation was obtained through a gentle palpation of the temples to detect dehydration. A score of 0 indicated a normal appearance, while increasing scores reflected greater severity, from slight sunken temples (1) to severe dehydration (4). The body condition of the subjects was evaluated based on various parameters, including growth, muscular development, coat quality, and the presence of signs of weakness or rickets. A score of 0 represented normal condition, with progressively higher scores (1–5) indicating poorer condition, from mild stunting (1) to severe emaciation and weakness (5). The respiratory health of the subjects was assessed through careful observation of coughing and breathing effort. Coughing was scored as 0 (absent), 1 (intermittent), or 2 (continuous), while breathing effort was scored as 0 (normal) or 1 (labored).

Two blood samples were collected individually by jugular vein on week 0 and 8, before the morning milk meal, using heparinized and non-heparinized tubes (BD Vacutainer; BD and Co., Franklin Lakes, NJ, USA). The tubes were immediately cooled in ice, then centrifuged at 3000 rpm for 15 min at 4 °C upon arrival at the laboratory. The plasma and serum were harvested and stored at −20 °C for further analysis. Plasma samples were analyzed to determine total protein (g/L), albumin (g/L), globulin (glob; g/L), albumin/globulin ratio (alb/glob ratio), oxidative stress (ROM; mmol H_2_O_2_/L), copper (µmol/L), zinc (µmol/L), plasma antioxidant protection (OXY; μmol HClO), urea (mmol/L), non-esterified fatty acids (NEFA; mmol/L), beta-hydroxybutyrate (BHB; mmol/L), glucose (mmol/L), total cholesterol (mmol/L), triglycerides (mmol/L), aspartate aminotransferase (AST; mmol/L), gamma-glutamyl transferase (GGT; IU/L), total bilirubin (µmol/L), creatine kinase (CK; IU/L), Ca (mmol/L), P (mmol/L), Mg (mmol/L), creatinine (µmol/L), high-density lipoprotein (HDL; mmol/L), and alkaline phosphatase (ALP) using enzymatic colorimetric methods validated under ISO/IEC 17025 accreditation [16], performed with a multiparametric clinical chemistry autoanalyzer (ILab 650; Instrumentation Laboratory Company, Lexington, MA, USA). Serum protein electrophoresis was performed to determine serum protein fractions, including α_1_-, α_2_-, β-, and γ-globulins (g/L), using an agarose gel electrophoresis system (Hydrasys, Sebia, Lisses, France).

The consistency of the feces was individually evaluated on a daily basis using a 4-point scale (0–3) that was designed to assess stool firmness. Scores of 0 and 1 indicated normal consistency, with 0 representing firm feces and 1 describing feces that retained their form but showed slight spreading. Scores of 2 and 3 reflected reduced fecal consistency: a score of 2 indicated soft feces that spread readily (mild diarrhea), whereas a score of 3 denoted liquid feces that splattered upon contact (severe diarrhea). Fecal color was evaluated at the same time as fecal consistency; scores ranged from 0 to 1, with 0 indicating brown feces and 1 indicating abnormal colors [15,17].

Fecal samples were collected from rectal ampulla, kept on ice, and subsequently frozen at –20 °C until analysis. Samples were collected on weeks 0, 4, and 8 from six calves per group, for the evaluation of bacterial load using the plate count method, as described by Dell’Anno et al. [18]. For the quantification of total bacteria load, coliforms and *Lactobacillus* spp. 1 g of feces was suspended in 9 mL of sterile physiological saline solution (0.9% NaCl) and homogenized. Serial 10-fold dilutions (10^−1^ to 10^−10^) were prepared, and 1 mL aliquots from appropriate dilutions were plated in triplicate on selective media: Plate Count Agar (PCA) for total bacteria, Violet Red Bile Lactose Agar (VRBLA) for coliforms, and De Man, Rogosa, and Sharpe agar (MRSA) for *Lactobacillus* spp. (Liofilchem, Teramo, Italy). Plates for total bacteria were incubated aerobically at 30 °C for 72 h, while *Lactobacillus* spp. and coliforms were incubated semi-anaerobically (using the inclusion and overlay method) at 30 °C and 40 °C for 72 h and 24 h, respectively. Colony-forming units (CFUs) were enumerated, and results were expressed as log_10_ CFU/g of feces, normalized to fecal dry matter content.

### 2.3. Statistical Analysis

Data was analyzed using R software (version 4.3.3). Data normality was assessed using the Shapiro–Wilk test. Animal performance, fecal bacterial counts, and blood metabolites were analyzed using the Aligned Rank Transform (ART) method (ARTool package, version 0.11.2, CRAN, 2025), which allows the application of factorial ANOVA to non-parametric data while preserving interaction effects. The statistical model included the fixed effects of treatment (Trt), time (Time), and their interaction (Trt × Time). Scores were evaluated by analyzing relative frequencies, and statistical significance was assessed using a Cumulative Link Mixed Model (CLMM), which is an ordinal regression model suitable for analyzing ordered categorical data. The model included the fixed effect of treatments (Trt), the effect of time (Time), the interaction between treatment and time (Trt × Time), and treatment and subgroups (Trt × Sub). Values were considered statistically different when *p* ≤ 0.050. All results are expressed as mean ± standard deviation (SD).

## 3. Results

### 3.1. Zootechnical Performance

Overall, body weight and average daily gain were not affected by dietary treatment. No treatment × subgroup interaction was observed (Table 2). Conversely, these outcomes exhibited a time-dependent increase over the course of the study, with BW, ADG, and ADFI progressively increasing throughout the experimental weeks (*p* < 0.001). No statistically significant treatment × time interaction was detected for body weight, although a numerical tendency was observed in the control group (*p* = 0.054). Overall, ADFI was not affected by treatment; however, in week 8 the CTR group showed the highest value (*p* = 0.036; CTR: 0.20 kg/d; TRT: 0.17 kg/d). Rectal temperature was not affected by dietary treatment (*p* > 0.05).

### 3.2. Clinical Scores

Clinical scores were not affected by the treatment (*p* > 0.05; Table 3). Time had a significant effect on the sunken eyes score (*p* < 0.05). A significant treatment × time interaction was detected, indicating that the effect of dietary treatment differed across the experimental period. Specifically, differences emerged during week 1; calves in the TRT group showed higher frequency compared with those in the CTR group (*p* = 0.029). Moreover, a significant treatment × subgroup interaction (*p* < 0.01) was also detected for sunken eyes. In particular, subgroup 1 (1–2 days of life) showed higher frequencies of critical scores (greater than 0) in TRT calves compared with CTR one (48.20% vs. 22.00%, respectively), whereas subgroup 3 (5–7 days of life) exhibited the opposite pattern, with higher frequencies in CTR relative to TRT calves (26.50% vs. 14.30%, respectively, Table 3).

Breathing effort was significantly influenced by time (*p* < 0.05), indicating a temporal evolution of respiratory clinical signs during the experimental period. However, no significant effects of treatment or treatment × time interaction were detected for this score.

Low body condition score was significantly affected by time (*p* < 0.05), and a significant treatment × time interaction was detected, indicating differential temporal patterns between treatments. Specifically, differences were observed during week 3, when TRT calves exhibited lower frequencies than CTR calves (*p* = 0.003). Subgroup analyses revealed additional variability. In subgroups 2 (3–4 days of life) and 3 (5–7 days of life), TRT calves showed lower frequencies of non-zero scores in week 3 (*p* = 0.001 and *p* = 0.003, respectively). In contrast, subgroup 1 (1–2 days of life) displayed higher frequencies of non-zero low body condition scores in TRT calves during week 4 (*p* = 0.008).

### 3.3. Blood Metabolic Profile

Blood parameters were differentially influenced by time, treatment and subgroup effects (Table 4, Table 5, Table 6, Table 7 and Table 8). Over time, calves showed a progressive maturation of metabolic status, characterized by increases in total protein and albumin, as well as in total cholesterol and HDL together with higher concentrations of selected trace minerals. In contrast, urea and bilirubin decreased over time. These changes are consistent with physiological development and improved metabolic efficiency. Among parameters related to passive immunity, total protein, albumin-to-globulin ratio, and globulin concentrations were significantly affected by the treatment × subgroup interactions detected for these variables (*p* < 0.05), whereas no main effect of treatment was observed. Although ARTool detected a significant treatment effect, post hoc comparisons at individual time points were not significant, likely due to reduced statistical power when data were stratified. The significant global effect indicates a consistent trend over time rather than differences in specific weeks. Gamma-glutamyl transferase, a marker of hepatic activity and colostrum-derived enzyme transfer, was significantly affected by treatment (*p* = 0.014), time (*p* < 0.01), and their interaction (*p* = 0.049). Although overall GGT concentrations tended to be lower in TRT compared with CTR calves, in week 8 GGT was significantly greater in TRT calves than in CTR (34.25 ± 33.25 vs. 23.67 ± 3.28 IU/L; *p* = 0.049). Creatinine was significantly affected by time (*p* < 0.01) and by the treatment × time interaction (*p* = 0.013). Creatinine showed a tendency toward a treatment × time interaction, with slightly higher values in TRT calves in week 8 (83.83 ± 11.46 vs. 81.42 ± 12.84 µmol/L; *p* = 0.069). Finally, mineral profile was influenced by time and treatment. In particular, calcium concentration was significantly affected by treatment (*p* < 0.01), being greater in TRT compared with CTR calves (2.86 ± 0.19 vs. 2.71 ± 0.20 mmol/L; *p* = 0.040). Phosphorus and copper were significantly affected by time, with copper also exhibiting a significant treatment × subgroup interaction (*p* = 0.004). Electrophoretic fractions also reflected age-related changes, with shifts in α_1_- and α_2_-globulins, while γ-globulin concentrations were significantly influenced by the treatment × subgroup interaction (*p* < 0.01).

### 3.4. Fecal Consistency, Color and Fecal Microbiology Analysis

Fecal characteristics were differentially influenced by time and treatment (Table 9). Fecal color was significantly affected by time, whereas fecal consistency was influenced by time, treatment × time interaction, and subgroup. The distribution of fecal color scores changed over the experimental period, with feces appearing more abnormal (yellow and green) during the first weeks of life and progressively shifting toward brownish coloration in later weeks, reflecting the transition toward normal fecal characteristics (*p* < 0.05).

**Figure 2:**
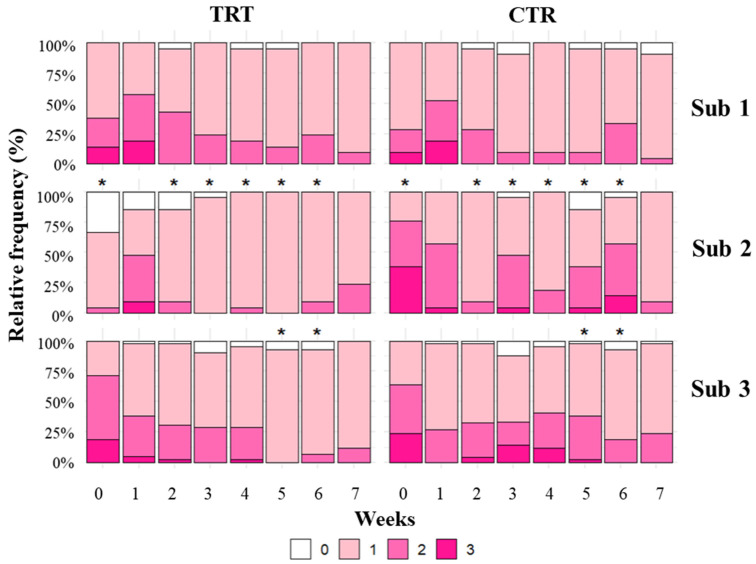
Relative frequency (%) of fecal consistency scores in treatment (TRT) and control (CTR) groups across subgroups (Sub 1, Sub 2, and Sub 3) and weeks. * Differences were considered statistically significant at *p* < 0.05 (Trt × Time). Fecal score 0–1 = normal feces, 2–3 = liquid feces (diarrhea).

Fecal consistency score was significantly lower in the TRT group compared with the CTR group within subgroup 2 (3–4 days of life), where the frequencies of diarrhea cases (score 2–3) were 12.5% vs. 39.3%, respectively (*p* < 0.001). Significant treatment × time interactions were observed in subgroups 2 (3–4 days of life) and 3 (5–7 days of life). In subgroup 2 (3–4 days of life), TRT calves exhibited lower fecal consistency scores during weeks 0, 2, 3, 4, 5, and 6 (*p* < 0.001, *p* = 0.042, *p* = 0.005, *p* = 0.006, *p* < 0.001, and *p* < 0.001, respectively), indicating a reduced frequency of fecal consistency scores greater than zero and, consequently, fewer diarrheic episodes. In subgroup 3 (5–7 days of life), a similar reduction was observed during weeks 5 and 6 (*p* = 0.051 and *p* = 0.050, respectively), confirming a time-dependent decrease in diarrhea incidence in treated calves (Figure 2).

Regarding fecal microbiology, total bacterial counts were not affected by treatment overall but decreased over time. In week 8, total bacterial counts were significantly lower in the CTR group compared with the TRT group (*p* < 0.01). In contrast, coliform bacteria and *Lactobacillus* spp. counts were significantly influenced by treatment and by the treatment × time interaction (Table 10).

Both bacterial groups, coliform bacteria and *Lactobacillus* spp., showed a decreasing trend over time and were consistently lower in the TRT group. Coliform counts were significantly lower in TRT calves in week 4 (3.82 ± 4 vs. 4.67 ± 4.5 log_10_ CFU/g; *p* < 0.001) and week 8 (4.37 ± 4.6 vs. 7.27 ± 7.54 log_10_ CFU/g; *p* < 0.001). Similarly, *Lactobacillus* spp. counts were lower in the TRT group compared with the CTR group in week 4 (6.04 ± 6.29 vs. 6.30 ± 6.57 log_10_ CFU/g; *p* = 0.041). Conversely, total bacterial counts in week 8 were higher in the TRT group than in the CTR group (7.70 ± 7.79 vs. 7.70 ± 7.60; *p* < 0.001).

## 4. Discussion

Astaxanthin supplementation administered through milk replacer resulted in a numerical tendency toward lower body weight in treated calves compared with controls, whereas average daily gain was not affected throughout the experimental period. The absence of differences in ADG indicates that growth rate was maintained despite minor differences in body weight, suggesting that astaxanthin supplementation did not impair overall growth performance. Body weight and average daily gain in young calves are closely associated with nutrient intake, and their relationship with average daily feed intake has been consistently reported in the literature [19,20]. Consistent with the present findings, several studies have reported no effects of astaxanthin supplementation on BW, or ADG in Murrah buffalo heifers [21] and in weaned pigs receiving *Hermetia illucens* larval meal combined with astaxanthin [8]. Astaxanthin did not affect growth performance in several species, including Murrah buffalo heifers [22], mice [23], laying hens [24], weaned pigs [8], and finishing pigs [25]. Similar neutral responses have also been observed in calves supplemented with carotenoids exhibiting antioxidant activity comparable to astaxanthin, such as β-carotene [26]. Only a limited number of studies have reported positive effects on growth performance, including heifers supplemented with astaxanthin [14], preweaning piglets born to supplemented sows [13], and aquaculture species [27], highlighting the species- and age-dependent nature of responses to astaxanthin on both growth performance and feed intake.

In the present study, differences in solid feed intake emerged only toward the end of the trial, when the CTR group showed a higher ADFI compared with TRT calves, without corresponding differences in ADG. Reductions in feed intake have been observed in finishing pigs and laying hens [24,25], whereas no effects on dry matter intake were reported in Murrah buffalo heifers supplemented with astaxanthin and in lactating cows supplemented with lutein, a structurally related xanthophyll [21,28].

In the present study, the maintenance of similar growth rates despite lower feed intake in the treated group may therefore indicate improved efficiency of nutrient utilization. However, nutrient efficiency was not directly measured in this study, and further studies should be considered, as no data are currently available in literature for preweaning dairy calves.

Astaxanthin is known to exert antioxidant and anti-inflammatory activity primarily through direct scavenging of reactive oxygen species; however, whether these effects translate into measurable changes in feed intake or growth performance appears to be highly context dependent [29,30].

Direct comparisons with Holstein preweaning calves remain limited, and differences in digestive physiology, dosage, and route of administration likely contribute to variability among studies. Nevertheless, several studies in the literature have investigated the use of feed additives derived from other algal species in calves, offering useful reference points for interpreting the present findings. For instance, supplementation with *Spirulina platensis* was associated with increased final body weight in growing Friesian calves [31], whereas supplementation with *Ascophyllum nodosum* did not affect body weight or average daily gain in preweaning calves [4].

To evaluate whether these growth and intake responses were associated with compromised health or physiological stress, rectal temperature and clinical health indicators were monitored throughout the experimental period. Rectal temperature did not differ among groups, indicating that astaxanthin supplementation did not induce systemic thermal stress and was well tolerated, particularly in light of the limited thermoregulatory capacity of neonatal calves [32]. Similarly, clinical scores assessing hydration status, respiratory function, and general welfare, including breathing effort, cough, sunken eyes, and body condition, were considered as functional indicators supporting the interpretation of growth and intake data [15]. Although sunken eyes and low body condition varied across subgroups and time points, these changes were consistent with the physiological vulnerability of the neonatal period and were not systematically associated with treatment, suggesting that astaxanthin did not adversely affect overall clinical status [33].

Further insights into the physiological responses to astaxanthin supplementation were provided by the serum metabolic profile. Among the analyzed parameters, treatment effects were detected for circulating calcium and gamma-glutamyl transferase. Higher circulating calcium concentrations observed in the TRT group could suggest a potential modulation of mineral homeostasis; however, calcium values remained within physiological ranges for preweaning calves and should be interpreted cautiously. Calcium plays a central role in gastrointestinal physiology in animals, contributing to smooth muscle contractility, gastric acid secretion, maintenance of epithelial barrier integrity, and immune signaling and is primarily absorbed in the small intestine, and its absorption efficiency declines progressively with advancing age [34,35]. However, astaxanthin has been reported to modulate intracellular Ca^2+^ handling under conditions of oxidative stress, suggesting a plausible mechanistic link between its antioxidant activity and calcium regulation [36].

Gamma-glutamyl transferase activity followed the expected age-related decline associated with intestinal maturation and reduced permeability to colostrum-derived enzymes [37]. Temporal differences in GGT activity between treatments and subgroups are therefore more likely to be attributable to variability in colostrum intake and early neonatal conditions rather than to a direct effect of astaxanthin supplementation. This interpretation is further supported by the absence of treatment effects on total bilirubin concentrations, which declined over time in both groups and remained within reference ranges, indicating no evidence of hepatic dysfunction [38]. The absence of treatment-related differences and the maintenance of bilirubin values within reference ranges suggest that astaxanthin supplementation did not adversely affect liver function or systemic health.

Time-dependent changes observed in α_1_- and α_2_-globulin fractions are consistent with immune maturation and resolution of early inflammatory responses during the neonatal period [39]. The absence of a clear treatment effect on total globulins or albumin-to-globulin ratio suggests that astaxanthin supplementation did not markedly influence passive immunity. Significant subgroup × treatment interactions detected for γ-globulins likely reflect variability in colostrum intake and early immune dynamics rather than a direct immunomodulatory effect of the dietary treatment.

The evaluation of different subgroups according to age at enrollment was useful to assess the effect of the additive across different starting days. No differences were observed in performance or hematological parameters; however, the improvement in fecal consistency score was more pronounced in specific subgroups. The TRT group showed reductions of 27% in subgroup 2 (3–4 days of life) and 8% in subgroup 3 (5–7 days of life), suggesting that the treatment’s effectiveness depends on the calf’s age at the time of supplementation (initiated between 4 and 6 days of life).

Notably, astaxanthin-supplemented calves exhibited a lower frequency of diarrhea episodes, representing the most biologically relevant functional outcome of the present study. This improvement in gastrointestinal health was not fully reflected by growth performance parameters alone and highlights the importance of considering health-related outcomes during the preweaning period. The reduction in diarrhea incidence was accompanied by changes in fecal bacterial populations, including lower coliform counts at mid- and late-experimental time points and higher total bacterial counts at the end of the trial. These findings suggest a restructuring of selected bacterial groups rather than a generalized antimicrobial effect. The role of gut microbiota in preweaning calf diarrhea is well established, as microbial imbalance, particularly the proliferation of enterotoxigenic *Escherichia coli*, has been associated with intestinal dysfunction and increased disease incidence, whereas a diversified and stable microbiota is indicative of gut health [40,41]. Accordingly, the reduction in coliforms observed in treated calves in weeks 4 and 8 is consistent with an improved intestinal environment. Astaxanthin may have contributed to these effects through indirect antibacterial actions against Gram-negative bacteria, including inhibition of biofilm formation, modulation of quorum sensing, and interference with bacterial metabolism, as previously reported in vitro and in vivo [42]. The concomitant increase in total bacterial counts further supports the hypothesis that astaxanthin promoted a reorganization of the gut microbiota rather than a generalized antimicrobial effect, potentially limiting pathogen-associated dysbiosis, reducing diarrhea incidence, and supporting stable growth despite lower feed intake. Astaxanthin has been shown to exhibit antibacterial properties against specific pathogenic microorganisms [42,43], which could in turn affect gut homeostasis, satiety signaling, and feeding behavior. However, further targeted studies are required to clarify the mechanisms underlying these interactions.

## 5. Conclusions

In the present study, astaxanthin supplementation administered through milk replacer did not affect growth performance in preweaning Holstein calves, as body weight and average daily gain were maintained throughout the experimental period. However, astaxanthin-supplemented calves exhibited a lower frequency of diarrhea episodes, which may suggest a beneficial effect on gastrointestinal health that was not fully captured by growth-related parameters alone. The reduction in diarrhea incidence was accompanied by changes in fecal bacterial populations, including lower coliform counts and higher total bacterial counts toward the end of the trial, which may indicate a modulation of selected components of the gut microbial environment rather than a generalized antimicrobial effect. Clinical health indicators and rectal temperature were not adversely affected by treatment, supporting the overall tolerability of astaxanthin supplementation during early life. Treatment-related differences observed in selected serum metabolic parameters, remained within physiological ranges and are more likely attributable to age-related developmental processes and early-life variability than to direct effects of astaxanthin. Overall, these findings suggest that astaxanthin supplementation could be associated with improved gastrointestinal health in preweaning calves without negatively affecting growth performance. Given the exploratory nature of the study, the limited sample size, and the reliance on culture-based microbiological methods, further research using larger cohorts and comprehensive microbiota and functional analyses is required to confirm these results and to elucidate the underlying mechanisms of action. Nevertheless, the reduction in diarrhea incidence and the associated changes in fecal bacterial populations provide promising evidence that astaxanthin supplementation may support gastrointestinal health in preweaning calves.

## Figures and Tables

**Table 1:** Nutritional composition of milk replacer (MR) and solid feed (SF; data provided by productor: UVL srl, Roè Volciano, Brescia, Italy).

Item	Milk Replacer ^1^ (% DM Basis)	Calf Starter ^2^ (% DM Basis)
Crude Protein	23	16.50
Ether Extract	23	2.50
Crude Fiber	0.1	12.50
Ash	6.8	5.10
Ca	1.02	not determined
P	0.65	not determined
Na	0.46	0.20

^1^ Additives per kg, nutritional additives: vitamin A 25,000 UI, vitamin D3 10,000 UI, vitamin E 100 mg, vitamin K3 5 mg, vitamin C 150 mg, vitamin B1 15 mg, potassium iodide 0.3 mg, Mn-sulfate monohydrate 55 mg, Zn-sulfate monohydrate 67 mg, Se-saccharomyces cerevisiae 0.2 mg, Fe-sulfate monohydrate II 60 mg technological integration: butylhidroxitoluene 35 mg. ^2^ Additives per kg, vitamins, provitamins, and substances with similar effects: vitamin A 23,000 UI, vitamin D3 2000, Biotin 0.20 mg, choline chloride 243.75 mg, niacinamide 61.50 mg, vitamin B1 2.45 mg, vitamin B2 0.60 mg, vitamin B6 0.75 mg, UI, vitamin E 25.00 mg vitamin K3 0.88 mg. Trace element compounds: copper sulfate pentahydrate—Cu 1.27 mg, potassium iodide 0.97 mg, iron(II) sulfate monohydrate—Fe 13.25 mg, manganese oxide—Mn 48.06 mg, sodium selenite—Se 0.14 mg, zinc oxide—18.75 mg.

**Table 2:** Calf performance in treatment (TRT) and control (CTR) groups across subgroups.

Items	TRT			CTR			*p* Value			
	Sub 1	Sub 2	Sub 3	Sub 1	Sub 2	Sub 3				
	Mean ± SD	Mean ± SD	Mean ± SD	Mean ± SD	Mean ± SD	Mean ± SD	Trt	Time	Trt × Time	Trt × Sub
BW	45.41 ± 9.8	50.38 ± 12.2	49.33 ± 12.6	50.45 ± 12.9	45.99 ± 11.4	52.61 ± 12.1	0.349	<0.01	0.054	0.083
ADG	0.41 ± 0.40	0.57 ± 0.37	0.56 ± 0.40	0.59 ± 0.40	0.52 ± 0.36	0.58 ± 0.37	0.143	<0.01	0.946	0.069
ADFI	0.18 ± 0.24	0.12 ± 0.12	0.21 ± 0.25	0.23 ± 0.25	0.15 ± 0.19	0.22 ± 0.26	0.309	<0.01	0.036	0.667

BW = body weight (kg); ADG = average daily gain (kg/d); ADFI = average daily feed intake (kg DMI/d). Sub indicates the subgroup, which is the same for each group. Subgroups were defined as follows: subgroup 1, calves aged 1–2 days; subgroup 2, calves aged 3–4 days; subgroup 3, calves aged 5–7 days. Results are expressed as mean ± standard deviation (SD). Differences were considered statistically significant at *p* < 0.05.

**Table 3:** Relative frequency (%) of clinical scores of calves in treatment (TRT) and control (CTR) groups across subgroups.

Items	Score	Freq (%)						*p* Value			
		TRT			CTR			Trt	Time	Trt × Time	Trt × Sub
		Sub 1	Sub 2	Sub 3	Sub 1	Sub 2	Sub 3				
	1	7.7	0.6	6.0	8.9	8.9	8.6				
Cough	0	97.6	98.2	95.5	97.6	97.6	96.1	0.981	0.993	0.954	0.912
	1	2.4	1.8	4.5	2.4	2.4	3.6				
	2	0.0	0.0	0.0	0.0	0.0	0.3				
Sunken eyes	0	51.8	91.1	85.7	77.4	82.7	72.9	0.180	<0.01	0.014	0.002
	1	42.9	8.3	13.4	19.0	14.9	22.3				
	2	5.4	0.0	0.9	3.0	2.4	4.2				
	3	0.0	0.6	0.0	0.6	0.0	0.6				
	4	0.0	0.0	0.0	0.0	0.0	0.0				
Low body condition	0	70.2	91.1	83.9	81.0	81.0	76.8	0.430	<0.01	0.027	0.108
	1	26.2	8.3	15.2	15.5	13.7	21.1				
	2	3.0	0.6	0.9	3.0	4.2	2.1				
	3	0.6	0.0	0.0	0.6	1.2	0.0				
	4	0.0	0.0	0.0	0.0	0.0	0.0				
	5	0.0	0.0	0.0	0.0	0.0	0.0				

Sub indicates the subgroup, which is the same for each group. Subgroups were defined as follows: subgroup 1, calves aged 1–2 days; subgroup 2, calves aged 3–4 days; subgroup 3, calves aged 5–7 days. Differences were considered statistically significant at *p* < 0.05.

**Table 4:** Metabolic profile parameters related to passive immunity transfer in calves from treatment (TRT) and control (CTR) groups across subgroups.

Items	TRT			CTR			*p* Value			
	Sub 1	Sub 2	Sub 3	Sub 1	Sub 2	Sub 3				
	Mean ± SD	Mean ± SD	Mean ± SD	Mean ± SD	Mean ± SD	Mean ± SD	Trt	Time	Trt × Time	Trt × Sub
Total protein (g/L)	62.2 ± 4.6	70.8 ± 10.5	64.8 ± 8.5	67.1 ± 4.3	60.4 ± 9.6	60.8 ± 8.9	0.112	<0.01	0.828	0.031
Alb/Glob (ratio)	1.2 ± 0.1	0.9 ± 0.4	1.0 ± 0.2	1.0 ± 0.3	1.1 ± 0.2	1.1 ± 0.2	0.615	0.084	0.525	0.043
Glob (g/L)	28.7 ± 2.5	38.2 ± 8.7	32.7 ± 6.4	33.4 ± 5.9	29.1 ± 5.5	29.0 ± 8.7	0.142	0.483	0.847	0.027
GGT (IU/L)	200 ± 185	312 ± 277	143 ± 172	606 ± 775	209 ± 249	144 ± 190	0.014	<0.01	0.049	0.001

Subgroups were defined as follows: subgroup 1, calves aged 1–2 days; subgroup 2, calves aged 3–4 days; subgroup 3, calves aged 5–7 days. Results are expressed as mean ± standard deviation (SD). Differences were considered statistically significant at *p* < 0.05. Alb/Glob = albumin/globulin, Glob = globulin, GGT = gamma-glutamyl transferase.

**Table 5:** Metabolic profile parameters related to energy metabolism in calves from treatment (TRT) and control (CTR) groups across subgroups.

Items	TRT			CTR			*p* Value			
	Sub 1	Sub 2	Sub 3	Sub 1	Sub 2	Sub 3				
	Mean ± SD	Mean ± SD	Mean ± SD	Mean ± SD	Mean ± SD	Mean ± SD	Trt	Time	Trt × Time	Trt × Sub
Glucose (mmol/L)	5.2 ± 1.0	5.5 ± 0.7	5.2 ± 0.7	6.5 ± 0.5	5.5 ± 1.4	5.1 ± 0.6	0.224	0.203	0.418	0.021
BHB (mmol/L)	0.11 ± 0.04	0.11 ± 0.05	0.09 ± 0.02	0.14 ± 0.03	0.09 ± 0.07	0.11 ± 0.04	0.255	<0.01	0.461	0.461
NEFA (mmol/L)	0.47 ± 0.13	0.43 ± 0.30	0.35 ± 0.07	0.48 ± 0.13	0.36 ± 0.15	0.45 ± 0.14	0.516	0.158	0.484	0.456
Creatinine (µmol/L)	95.5 ± 15.2	104.0 ± 21.6	102.0 ± 43.2	97.8 ± 102.0	93.8 ± 57.1	130.8 ± 18.7	0.217	<0.01	0.013	0.255
CK, IU/L	178 ± 154	141 ± 119	166 ± 81	217 ± 65	149 ± 131	172 ± 107	0.694	<0.01	0.126	0.507
Urea (mmol/L)	5.57 ± 2.12	5.17 ± 2.25	5.80 ± 2.33	6.47 ± 5.47	5.82 ± 3.64	6.9 ± 1.9	0.290	<0.01	0.944	0.943

Subgroups were defined as follows: subgroup 1, calves aged 1–2 days; subgroup 2, calves aged 3–4 days; subgroup 3, calves aged 5–7 days. Results are expressed as mean ± standard deviation (SD). Differences were considered statistically significant at *p* < 0.05. BHB = beta-hydroxybutyrate, NEFA = non-esterified fatty acids, CK = creatine kinase.

**Table 6:** Metabolic profile parameters related to liver functionality in calves from treatment (TRT) and control (CTR) groups across subgroups.

Items	TRT			CTR			*p* Value			
	Sub 1	Sub 2	Sub 3	Sub 1	Sub 2	Sub 3				
	Mean ± SD	Mean ± SD	Mean ± SD	Mean ± SD	Mean ± SD	Mean ± SD	Trt	Time	Trt × Time	Trt × Sub
Albumin (g/L)	33.4 ± 3.4	32.5 ± 3.1	32.1 ± 3.5	33.7 ± 2.2	31.0 ± 5.1	32.0 ± 3.4	0.790	<0.01	0.887	0.481
Total cholesterol (mmol/L)	2.2 ± 2.3	2.2 ± 1.2	2.5 ± 1.1	2.5 ± 1.4	2.2 ± 1.9	2.4 ± 1.5	0.864	<0.01	0.627	0.755
Triglyceride (mg/dL)	0.31 ± 0.07	0.25 ± 0.06	0.21 ± 0.17	0.23 ± 0.05	0.21 ± 0.07	0.26 ± 0.10	0.852	0.674	0.260	0.244
HDL, (mmol/L)	1.3 ± 1.0	1.3 ± 0.8	1.5 ± 0.6	1.4 ± 0.7	1.3 ± 1.0	1.4 ± 0.8	0.648	<0.01	0.376	0.736
ALP (IU/L)	258 ± 127	196 ± 88	255 ± 30	295 ± 66	226 ± 126	258 ± 70	0.532	0.0558	0.684	0.774
AST, (mmol/L)	54.2 ± 15.0	40.2 ± 9.5	47.0 ± 10.1	56.3 ± 9.8	41.7 ± 13.2	43.3 ± 9.0	0.514	0.063	0.741	0.693
Total Bilirubin (µmol/L)	12.5 ± 5.7	7.6 ± 12.8	5.2 ± 5.1	7.5 ± 3.4	6.3 ± 8.9	7.5 ± 3.9	0.549	<0.01	0.072	0.072

Subgroups were defined as follows: subgroup 1, calves aged 1–2 days; subgroup 2, calves aged 3–4 days; subgroup 3, calves aged 5–7 days. Results are expressed as mean ± standard deviation (SD). Differences were considered statistically significant at *p* < 0.05. HDL = high-density lipoprotein, ALP = alkaline phosphatase, AST = aspartate aminotransferase.

**Table 7:** Metabolic profile parameters related to oxidative stress, mineral status, and inflammatory response in calves from treatment (TRT) and control (CTR) groups across subgroups.

Items	TRT			CTR			*p* Value			
	Sub 1	Sub 2	Sub 3	Sub 1	Sub 2	Sub 3				
	Mean ± SD	Mean ± SD	Mean ± SD	Mean ± SD	Mean ± SD	Mean ± SD	Trt	Time	Trt × Time	Trt × Sub
ROM (mmolH_2_O_2_/L)	1.3 ± 0.6	1.9 ± 0.8	2.0 ± 0.7	1.4 ± 0.7	1.7 ± 0.8	2.3 ± 0.8	0.673	0.113	0.093	0.562
OXY (μmol HClO)	387 ± 66	409 ± 73	418 ± 50	393 ± 46	365 ± 43	420 ± 53	0.571	0.649	0.261	0.227
Calcium (mmol/L)	2.9 ± 0.2	3.0 ± 0.2	2.8 ± 0.2	3.0 ± 0.2	2.6 ± 0.3	2.6 ± 0.2	<0.01	0.500	0.432	0.008
Phosphorus (mmol/L)	2.7 ± 0.8	2.5 ± 0.5	2.7 ± 0.4	2.8 ± 0.3	2.6 ± 0.6	2.7 ± 0.3	0.630	<0.01	0.966	0.694
Magnesium (mmol/L)	0.9 ± 0.1	0.9 ± 0.1	0.9 ± 0.1	1.0 ± 0.1	1.0 ± 0.1	0.9 ± 0.1	0.107	0.156	0.565	0.548
Copper (µmol/L)	7.5 ± 1.9	10.0 ± 7.7	10.1 ± 3.0	10.1 ± 2.0	8.4 ± 2.8	9.2 ± 2.3	0.696	<0.01	0.948	0.004
Zinc (µmol/L)	11.6 ± 2.1	13.7 ± 3.2	12.7 ± 3.3	12.0 ± 2.8	12.8 ± 2.8	13.0 ± 2.3	0.933	<0.01	0.884	0.836

Subgroups were defined as follows: subgroup 1, calves aged 1–2 days; subgroup 2, calves aged 3–4 days; subgroup 3, calves aged 5–7 days. Results are expressed as mean ± standard deviation (SD). Differences were considered statistically significant at *p* < 0.05.

**Table 8:** Metabolic profile parameters related to electrophoretic fractions in calves from treatment (TRT) and control (CTR) groups across subgroups.

Items	TRT			CTR			*p* Value			
	Sub 1	Sub 2	Sub 3	Sub 1	Sub 2	Sub 3				
	Mean ± SD	Mean ± SD	Mean ± SD	Mean ± SD	Mean ± SD	Mean ± SD	Trt	Time	Trt × Time	Trt × Sub
α_1_-globulin (g/L)	8.0 ± 3.2	7.3 ± 2.0	7.5 ± 3.0	8.2 ± 2.7	7.1 ± 2.7	7.2 ± 1.8	0.617	<0.01	0.530	0.911
α_2_-globulin (g/L)	2.8 ± 1.0	3.3 ± 1.2	3.5 ± 0.9	3.1 ± 1.1	3.0 ± 1.5	3.3 ± 1.8	0.437	<0.01	0.346	0.470
β-globulin (g/L)	6.0 ± 1.4	7.3 ± 1.0	7.5 ± 1.9	6.8 ± 1.4	6.62 ± 1.6	7.3 ± 1.6	0.890	0.654	0.541	0.443
γ-globulin (g/L)	8.4 ± 2.6	15.4 ± 3.7	12.1 ± 3.6	12.2 ± 5.0	10.3 ± 2.8	10.1 ± 6.5	0.460	0.658	0.398	0.001

Subgroups were defined as follows: subgroup 1, calves aged 1–2 days; subgroup 2, calves aged 3–4 days; subgroup 3, calves aged 5–7 days. Results are expressed as mean ± standard deviation (SD). Differences were considered statistically significant at *p* < 0.05.

**Table 9:** Relative frequency (%) of fecal scores of calves in treatment (TRT) and control (CTR) groups across subgroups.

Items	Score	Freq (%)						*p* Value			
		TRT			CTR			Trt	Time	Trt × Time	Trt × Sub
		Sub 1	Sub 2	Sub 3	Sub 1	Sub 2	Sub 3				
Fecal color	0	33.3	24.4	33.0	38.7	29.8	33.3	0.714	<0.01	0.073	0.982
	1	66.7	75.6	67.0	61.3	70.2	66.7				
Fecal consistency	0	1.8	8.3	4.2	4.2	3.0	4.2	0.992	<0.01	0.019	0.010
	1	69.6	79.2	68.8	73.8	57.7	61.0				
	2	24.4	11.3	23.5	18.5	31.0	27.7				
	3	4.2	1.2	3.6	3.6	8.3	7.1				

Sub indicates the subgroup, which is the same for each group. Subgroups were defined as follows: subgroup 1, calves aged 1–2 days; subgroup 2, calves aged 3–4 days; subgroup 3, calves aged 5–7 days. Differences were considered statistically significant at *p* < 0.05.

**Table 10:** Fecal bacterial count in total bacteria (log_10_ CFU/g), coliform bacteria (log_10_ CFU/g) and *Lactobacillus* spp. (log_10_ CFU/g) in week 0, 4, 8 in treatment (TRT) and control (CTR) groups.

Items	TRT			CTR			*p* Value		
	0	4	8	0	4	8			
	Mean ± SD	Mean ± SD	Mean ± SD	Mean ± SD	Mean ± SD	Mean ± SD	0	4	8
Total bacteria	8.7 ± 9.0	9.23 ± 9.4	7.70 ± 7.6	9.0 ± 9.1	7.1 ± 7.2	7.7 ± 7.6	0.619	0.763	<0.001
Coliform bacteria	7.8 ± 7.9	3.82 ± 4.0	4.37 ± 4.6	7.6 ± 7.9	4.7 ± 4.5	7.3 ± 7.5	0.463	<0.001	<0.001
*Lactobacillus* spp.	8.3 ± 8.0	6.04 ± 6.2	5.69 ± 5.7	7.5 ± 7.7	6.3 ± 6.6	7.4 ± 7.4	0.894	0.041	0.877

Results are expressed as mean ± standard deviation (SD). Differences were considered statistically significant at *p* < 0.05.

## Data Availability

The data that supports the findings of this study are available from the corresponding author upon reasonable request.

## Abbreviations

The following abbreviations are used in this manuscript:

ADFI	Average Daily Feed Intake.
ADG	Average Daily Gain.
ALP	Alkaline Phosphatase.
ARTool	Aligned Rank Transform Tool.
AST	Aspartate Aminotransferase.
BHB	Beta-Hydroxybutyrate.
BW	Body Weight.
Ca	Calcium.
CFU	Colony-Forming Units.
CK	Creatine Kinase.
CLMM	Cumulative Link Mixed Model.
CTR	Control Group.
DM	Dry Matter.
GGT	Gamma-Glutamyl Transferase.
Glob	Globulin.
HDL	High-Density Lipoprotein.
MR	Milk Replacer.
NEFA	Non-Esterified Fatty Acids.
OXY	Plasma Antioxidant Protection.
P	Phosphorus.
PCA	Plate Count Agar.
ROM	Reactive Oxygen Metabolites.
RT	Rectal Temperature.
SD	Standard Deviation.
SF	Solid Feed.
Sub	Subgroup.
TRT	Treatment Group.
VRBLA	Violet Red Bile Lactose Agar.
